# Expression and characterisation of human glycerol kinase: the role of solubilising agents and molecular chaperones

**DOI:** 10.1042/BSR20222258

**Published:** 2023-04-21

**Authors:** Riva Mary Rani, Superior Syngkli, Joplin Nongkhlaw, Bidyadhar Das

**Affiliations:** Biological Chemistry Laboratory, Department of Zoology, North-Eastern Hill University, Shillong 793022, India

**Keywords:** glycerol kinase, molecular chaperones, sarkosyl, solubilising agents, Type 2 diabetes mellitus

## Abstract

Background: Glycerol kinase (GK; EC 2.7.1.30) facilitates the entry of glycerol into pathways of glucose and triglyceride metabolism and may play a potential role in Type 2 diabetes mellitus (T2DM). However, the detailed regulatory mechanisms and structure of the human GK are unknown.

Methods: The human GK gene was cloned into the pET-24a(+) vector and over-expressed in *Escherichia coli* BL21 (DE3). Since the protein was expressed as inclusion bodies (IBs), various culture parameters and solubilising agents were used but they did not produce bioactive His-GK; however, co-expression of His-GK with molecular chaperones, specifically pKJE7, achieved expression of bioactive His-GK. The overexpressed bioactive His-GK was purified using coloumn chromatography and characterised using enzyme kinetics.

Results: The overexpressed bioactive His-GK was purified apparently to homogeneity (∼295-fold) and characterised. The native His-GK was a dimer with a monomeric molecular weight of ∼55 kDa. Optimal enzyme activity was observed in TEA buffer (50 mM) at 7.5 pH. K^+^ (40 mM) and Mg^2+^ (2.0 mM) emerged as prefered metal ions for His-GK activity with specific activity 0.780 U/mg protein. The purified His-GK obeyed standard Michaelis–Menten kinetics with *K*_m_ value of 5.022 µM (*R*^2^=0.927) for its substrate glycerol; whereas, that for ATP and PEP was 0.767 mM (*R*^2^=0.928) and 0.223 mM (*R*^2^=0.967), respectively. Other optimal parameters for the substrate and co-factors were also determined.

Conclusion: The present study demonstrates that co-expression of molecular chaperones assists with the expression of bioactive human GK for its characterisation.

## Introduction

Glycerol kinase (GK; EC 2.7.1.30) belongs to the FGGY carbohydrate kinase family (ATP: glycerol 3-phosphotransferase) that transfers a phosphate group from ATP to glycerol. GK − the key enzyme in the regulation of glycerol uptake and its metabolism − was first isolated from *E. coli* and studied by Cozzarelli and Lin [1]. The enzyme acts as a catalyst in the Mg^2+^-ATP-dependent phosphorylation of glycerol to yield G3P, which is a crucial intermediate in various metabolic pathways, such as the synthesis of glycerolipids and triglycerides, glycogenesis, glycolysis and gluconeogenesis [2]. The *E. coli* GK gene is only 48% homologous to that of human GK [3] and can act as a dimer and a tetramer, whereas, mammalian GK acts as a dimer [4]. The GK gene family comprises five loci, which are located on the X chromosome (Xp and Xq) and chromosome 4 [5,6]. The Xp21.3 GK is responsible for the X-linked disorder (GKD) and is composed of 21 exons extending over 50 kb [7,8]. The other loci are without introns, three of the genes are expressed and one gene is a non-coding pseudogene [6].

GK is involved in the glycerol 3-phosphate shuttle and, in combination with glycerol 3-phosphate dehydrogenase, forms dihydroxyacetone phosphate from glycerol [9]. In mammalian tissues, glycerol is derived from either synthesis by glyceroneogenesis or hydrolysis of triacylglycerols. Glycerol in the cells is phosphorylated by GK using ATP to form G3-P, which is transferred to various metabolic pathways, sustaining an incoming glycerol flow gradient. GK is instrumental in plasma glycerol withdrawal, utilization of glycerol by different tissues, and in carbohydrate homeostasis [10]. GK is present at the interface of lipid- and carbohydrate-metabolism and has been implicated in T2DM [11] and in disrupted lipid metabolism [12]. GK knockout mice were observed to have reduced glucose uptake [13], while individuals with GKD develop either T2DM or impaired glucose tolerance with obesity as one of the complex traits [14]. GK stimulates glycerol incorporation into triglyceride and reduces free fatty acid secretion from adipocytes. The expression of GK in adipocytes contributes to a reduction in the amount of free fatty acid levels and insulin sensitization by antidiabetic therapies [15]. This demonstrates that GK has a potential role in the treatment of T2DM. However, the detailed regulatory mechanisms of GK and its crystal structure have not yet been resolved. Therefore, the present study aimed to overexpress and characterise the human form of the enzyme. The GK gene was synthesized and cloned into a pET-24a(+) vector and over-expressed in *E. coli* BL21 (DE3). The recombinant protein was expressed as IBs; thus, various parameters were trialled to solubilise the protein for its subsequent characterisation. Human GK was finally over-expressed along with molecular chaperones and was purified and characterised.

## Materials and methods

### Chemicals and vectors

Glycerol_kinase_pET-24a(+) Plasmid, was procured from GenScript, U.S.A. QIAprep Spin Miniprep Kit (27104) was obtained from Qiagen, Germany. IPTG (I5502), kanamycin (K1876), L-Glutathione reduced (4251) and L-Glutathione oxidized (4501) were acquired from Sigma, U.S.A. Bacterial culture media such as yeast extract powder (RM027) and tryptone (CR014) were procured from Himedia Laboratories, India. N-Lauroylsarcosine sodium salt (L9150), L-arginine monohydrochloride (A5131), CTAB (H5882), guanidine hydrochloride (G3272), CHAPS hydrate (C9426) and glycylglycine (G0674) were procured from Sigma, U.S.A. A chaperone plasmid set (3340) was ordered from Takara, Japan. Amicon Ultra-15 centrifugal filters (UFC901008) were purchased from Millipore, U.S.A. HisTrap HP column (5×5 mL, 17524802), HiTrap desalting column (5×5 mL, 17140801) and HiPrep™ 16/60 Sephacryl™ S-200 HR (17116601) were procured from Cytiva, U.S.A. TEA hydrochloride (80184), PEP (40083), ATP (84878), glycerol (G5516), NADH (77268), LDH (49363) and PK (29986) were supplied by Sisco Research Limited, India. Other general chemicals and reagents were procured from local vendors.

### Codon optimisation of GK for heterologous expression in *E. coli*

For heterologous expression of the human GK gene (GenBank accession no. L13943.1) in *E. coli*, the codons for human GK amino acids were synthesized and optimised using Optimum GeneTM algorithm (GenScript, U.S.A.), which optimizes a variety of parameters that are critical to the efficiency of gene expression in *E. coli*. The optimised GK gene sequence was cloned into the pET-24 a(+) vector for over-expression.

### Active site prediction and protein alignment

The amino acids in the active site of GK were analysed from the crystal structure in Protein Data Bank (PDB ID: 1GLF) by evaluating the interactions of ligands (glycerol, ADP and PO_4_^−^) with the amino acids of GK (https://www.rcsb.org/). To identify similarities of the synthesised and optimised human GK amino acids with other available amino acids sequences of GK, the amino acids sequences from different organisms including humans [GenBank accession no. CAA55365.1 (*Homo sapiens*), AAC52824.1 (*Mus musculus*), NP_001108056.1 (*Danio rerio*), GFP68323.1 (*Saccharomyces cerevisiae*), NP_494721.1 (*Caenorhabditis elegans*), BAI79241.1 (*Trypanosoma brucei gambiense*) and EDV66601.1 (*Escherichia coli*)] were accessed from the NCBI database and aligned using the Clustal omega web server (https://www.ebi.ac.uk/Tools/msa/clustalo/). Clustal omega [16] was run with default parameters over the entire range (ClustalW with character counts).

### Cloning and overexpression of His-GK

The synthesised GK gene was cloned into a pET24-a(+) vector and transformed into the *E. coli* (DH5α) cells using heat shock method to generate the plasmid. Following cell growth, the plasmid was extracted using a miniprep kit following the manufacturer’s procedure. For expression of the recombinant protein, the isolated His**-**GK construct was then transformed into *E. coli* (BL21) cells using the heat shock method. A single colony of *E. coli* (BL21) was collected from the LB agar plate and inoculated in 10 ml LB broth supplemented with 50 µg/ml kanamycin. The culture was incubated overnight at 37°C with continuous shaking at 180 rpm using an Excella E25 shaking incubator (New Brunswick Scientific) overnight. Subsequently, 10 ml of the overnight culture was inoculated into 1 L LB medium and incubated at 37°C for 3 h. The bacterial cells were then induced with 0.5 mM IPTG and cultured overnight at 24°C. The following day, bacterial cells were harvested by centrifugation at 6,500 rpm, re-suspended in buffer A [Tris-HCl (pH 7.0), 1.0 mM EDTA, 2.0 mM DTT, 0.1 mM PMSF] and lysed by sonication for 5 min (10 s pulse ON and 50 s pulse OFF cycle at 25% amplitude). The fractions (supernatant and pellet) were analysed by 10% SDS-PAGE and the protein bands were visualized using a GelDoc system (UVP GelDoc-It 310, UVP Ltd, U.K.).

Since His-GK was expressed as IBs, various culture parameters, such as different concentrations of IPTG (0.2, 0.5 and 1.0 mM), induction periods (2, 4, 6 and 16 h), induction temperatures (16, 20, 24 and 37°C), culture media (LB, TB, auto-induction media) and additives (glycylglycine, glycerol, ethanol), were trialled to express soluble protein from *E. coli* (*in vivo*).

### Analysis of proteins by SDS-PAGE and native-PAGE

The protein fractions were analysed by 10% SDS-PAGE and gels were stained using 0.25% Coomassie Brilliant Blue (R250) as described by Green and Sambrook [17]. The de-stained SDS-PAGE gel was documented using a UVP GelDoc-It 310, UVP Ltd, U.K. Native-PAGE (without the denaturants used in SDS-PAGE) was also run for determination of protein subunits (monomer or oligomers) following the above-mentioned method [17].

### Solubilisation of recombinant His-GK from IBs and refolding

To solubilise the recombinant protein from IBs, several solubilising agents (DMSO, GdnHCl, urea and sarkosyl), a zwitterionic detergent (CHAPS), a cationic detergent (CTAB) and the amino acid arginine were used in vitro. Briefly, the cell pellet was resuspended in ice-cold buffer A and sonicated. The lysate was then centrifuged at 10,000 rpm for 30 min at 4°C to collect the IBs. The pellet containing the IBs was washed twice in ice-cold water containing 2% Triton X-100, followed by one wash in Tris-HCl buffer (pH 7.4) without Triton X-100. The washed IBs were then resuspended in buffer A containing the respective solubilising agent at a specific concentration and were incubated overnight at 4°C. The solubilised His-GK was centrifuged at 10,000 rpm for 30 min and the protein fractions were analysed by 10% SDS-PAGE.

Solubilization treatment with 8.0 M urea results in denaturation of the protein. Therefore, a stepwise dialysis method [18] involving gradual removal of urea (6.0, 3.0, 2.0, 1.0 and 0.5 M) was performed to refold the denatured protein. Briefly, dialysis of the sample was performed at 4°C against 100-fold buffer comprising Tris-HCl (pH 7.4), 2 mM DTT and the gradually decreasing urea concentration (as mentioned above) with continuous stirring. GSSG (1.0 M) and GSH (10.0 M) were added into the dialysis buffer at the 1.0 and 0.5 M urea stages only. The respective buffers were changed every hour and the sample was dialysed overnight at 4°C in Tris-HCl (pH 7.4) and 2 mM DTT buffer.

### Co-expression of His-GK with molecular chaperones

Chaperone plasmids (pG-KJE8, pGro7, pKJE7, pG-Tf2 and pTf16) were transformed individually into *E. coli* (BL21) cells. The His-GK construct was then transformed into *E. coli* (BL21) cells containing the respective chaperone plasmid. Transformed bacteria cells were cultured in 1 L LB medium with 20 μg/ml chloramphenicol and 50 μg/ml kanamycin. After incubation for 2 h, the respective chaperone induction reagents were added to the cultures; 0.5 mg/ml L-arabinose for the chaperones pGro7, pKJE7, and pTf16, 5 ng/ml tetracycline for pG-Tf2 and 0.5 mg/ml L-arabinose and 5 ng/ml tetracycline for pG-KJE8. Subsequently, 1 h after the induction of chaperones, expression of His-GK was induced from the bacteria by the addition of 0.5 mM IPTG and induction overnight at 24°C. Bacteria were harvested, re-suspended in lysis buffer A, and sonicated as described above. The cell lysate was centrifuged at 10,000 rpm for 30 min and the protein fractions were analysed by 10% SDS-PAGE.

### Purification of recombinant His-GK

Over-expressed recombinant His-GK was purified by HisTrap HP column using an AKTA Start Protein Purification System, (Cytiva, U.S.A). Initially, the column was equilibrated using 10 column volumes of binding buffer {50 mM Tris-HCl (pH 7.4), 10 mM imidazole, 300 mM NaCl and 2 mM DTT}. A 10% lysate was prepared as described in the ‘cloning and overexpression of His-GK’ section and was passed through the column. Unbound proteins were removed by washing with five column volumes of binding buffer. The bound protein was then eluted using 5 ml of elution buffer [50 mM Tris-HCl (pH 7.4), 300 mM imidazole and 2 mM DTT].

Desalting of the eluted protein was performed using a HiTrap desalting column with an AKTA Pure protein purification system (Cytiva-U.S.A). An Amicon Ultra centrifugal concentrator (30 K) was used to concentrate the eluted protein to <2.0 ml. The column was equilibrated with five column volumes of desalting buffer [50 mM Tris-HCl buffer (pH 7.4), and 2.0 mM DTT]. The protein was passed through the column and was subsequently eluted by the same buffer. The eluted protein was concentrated to <2.0 ml using an Amicon Ultra centrifugal 30 K concentrator and used for SEC.

To attain the highest form of purified target protein, SEC was performed using an SEC column (HiPrep™ 16/60 Sephacryl™ S-200 HR) with the Cytiva-AKTA Pure protein purification system, U.S.A. The SEC column was equilibrated with five column volumes of buffer [50 mM Tris-HCl (pH 7.4) and 2.0 mM DTT]. The desalted concentrated protein was loaded onto the column and subsequently eluted by one column volume of the buffer. The fractions of eluted protein were collected and stored at −20°C and enzyme kinetics were performed the next day. For longer storage, the proteins were concentrated (2 mg/ml), snap-frozen in liquid nitrogen, and stored at −80°C. All the purification processes were performed at 4°C.

### Enzyme kinetics and Michaelis–Menten constants

Enzyme kinetics were performed following a modified method of Krakow and Wang [19]. All assays were conducted at 37°C and oxidation of NADH was monitored at 340 nm in a Cary 60 UV-Vis spectrophotometer (Agilent Technologies, U.S.A.) fitted with a Peltier temperature-controlled system. The enzyme kinetics were performed in 1 mL of reaction mixture containing 75 mM TEA buffer (pH 7.4), 37 mM KCI, 10 mM MgCl_2_, 2 mM PEP, 0.5 mM NADH, 2 mM ATP, 5.5 mM glycerol, PK (10 U) and LDH (20 U). The assay mixture was pre-incubated for 5 min, then 20 µl of the purified enzyme from the SEC column was added to start the reaction. Enzyme activities were determined by taking 6.22 × 10^6^ as the molar extinction coefficient value for NADH. Bradford’s reagent was used to determine the total protein content taking BSA as the standard [20]. One unit of enzyme activity is defined as the amount of the enzyme that catalyses the oxidation of 1 μmol NADH per min under standard assay conditions. Specific activity was calculated and denoted as U/mg of protein.

To determine the optimal His-GK activity, several buffers (TEA, imidazole, Tris-HCl, PBS, HEPES), pH (6.5, 7.0, 7.5, 8.0, 8.5, 9.0, 9.5 and 10.0), metal ions (CaCl_2_, KCl, LiCl, MgCl_2_ and NaCl) and concentrations of Mg^2+^ (0.6, 0.8, 1.0, 2.0, 5.0 and 10.0 mM), PEP (0.1, 0.2, 0.4, 0.6, 0.8, 1.0 and 2.0 mM), glycerol (1, 2, 4, 6, 8, 10, 20 and 50 µM) and ATP (0.2, 0.4, 0.6, 0.8, 1.0 and 2.0 mM) were evaluated. Kmapp for the substrate (glycerol) and other co-factors (PEP and ATP) were determined by plotting the reaction rates at varying concentrations of the substrate and co-factors (Michaelis–Menten, Lineweaver–Burk and Eadie–Hofstee plots).

### Statistical analysis

The data were obtained from at least three individual experiments and were statistically analysed and presented as mean±SEM. Kmapp and Vmax were calculated using SigmaPlot 14.5 software and the positive correlation was not less than 0.9 (*R*^2^ >0.9).

## Results

### Analysis of GK active site

**Figure 1:**
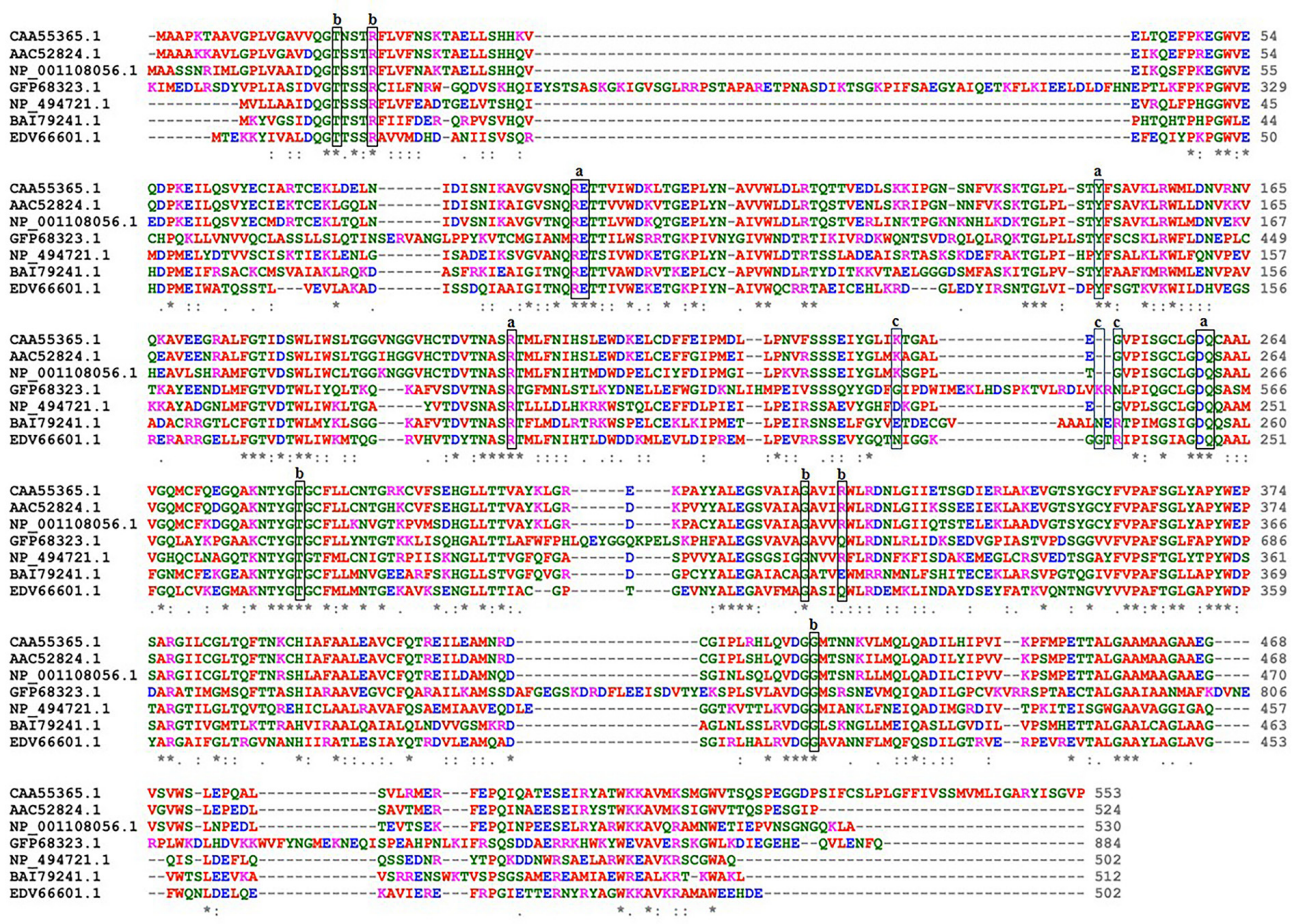
Alignment of amino acids sequences of GK The amino acids sequences of GK were accessed from NCBI (https://www.ncbi.nlm.nih.gov/) and aligned using CLUSTAL O (1.2.4) multiple sequence alignment program (https://www.ebi.ac.uk/Tools/msa/clustalo): GenBank accession no. CAA55365.1 (*Homo sapiens*), AAC52824.1 (*Mus musculus*), NP_001108056.1 (*Danio rerio*), GFP68323.1 (*Saccharomyces cerevisiae*), NP_494721.1 (*Caenorhabditis elegans*), BAI79241.1 (*Trypanosoma brucei gambiense*) and EDV66601.1 (*Escherichia coli*). The amino acids in the rectangles are involved in the active sites of GK; interaction with glycerol are marked as ‘a’, ADP as ‘b’ and with phosphate ion as ‘c’. ‘*’ indicates as identical amino acid residues; ‘:’ as conserved residues; ‘.’ as semi conserved residues; ‘-’ as absence of amino acid in the sequence.

The codon optimised human GK amino acids sequence, which was required for heterologous expression in *E. coli*, was aligned along with the available GK amino acids sequences from different organisms obtained from NCBI to identify the similarities between the amino acids sequences of GK. The alignments showed that the amino acids present in the active site, as observed from crystal analysis (PDB ID: 1GLF), were identical with the amino acids of the codon optimised GK (Figure 1).

### Optimisation of His-GK overexpression

**Figure 2:**
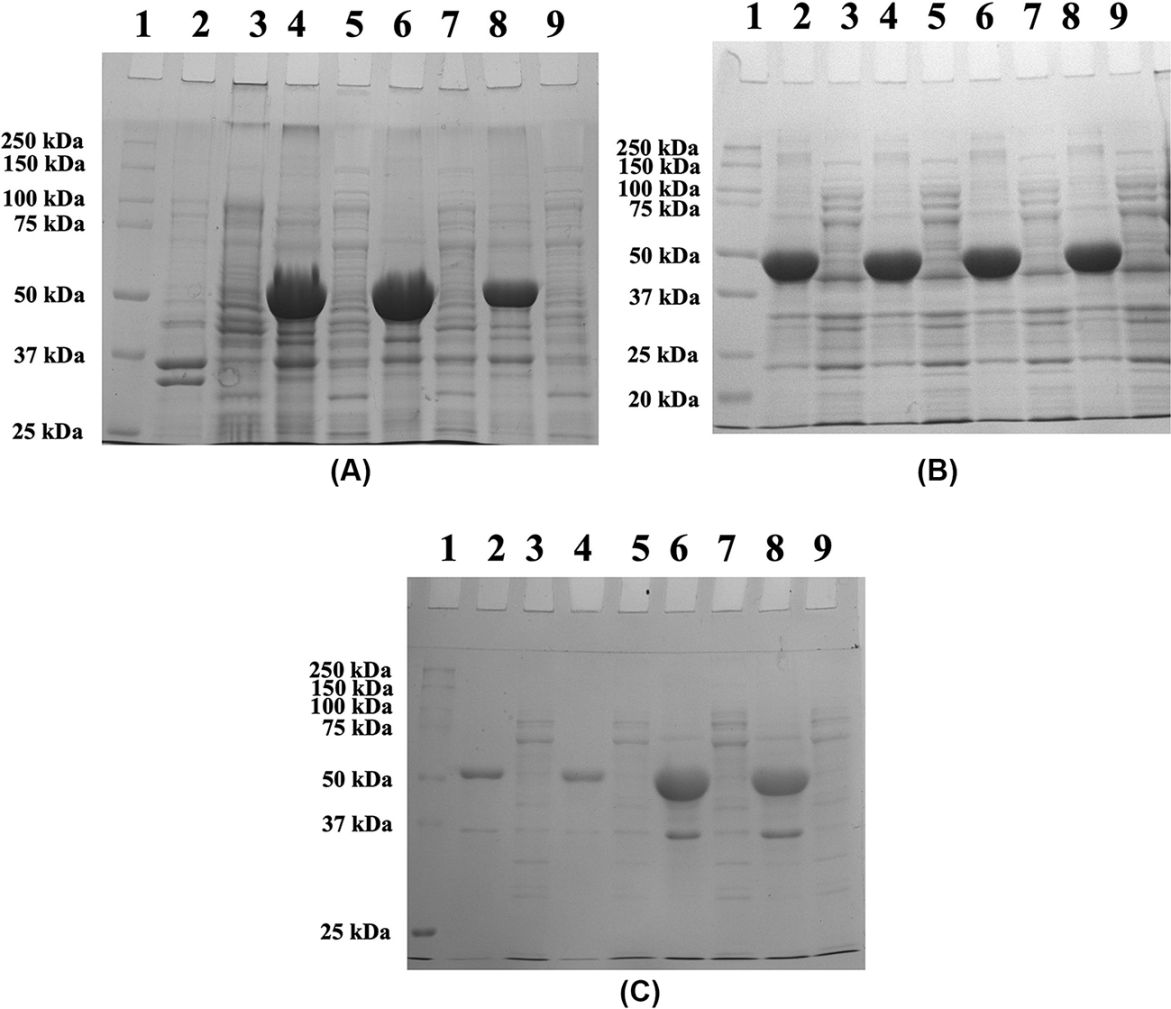
Effect of IPTG concentrations, temperatures, and induction periods on His-GK solubility (**A**) His-GK was induced in *E. coli* (BL21) with various concentrations of IPTG overnight at 37°C, and the proteins were analysed by 10% SDS-PAGE. Lane 1 - Marker; Lane 2 - Pellet (uninduced control); Lane 3 - Supernatant (uninduced control); Lane 4 - Pellet (1.0 mM IPTG); Lane 5 - Supernatant (1.0 mM IPTG); Lane 6 - Pellet (0.5 mM IPTG); Lane 7 - Supernatant (0.5 mM IPTG); Lane 8 - Pellet (0.2 mM IPTG); Lane 9 - Supernatant (0.2 mM IPTG). (**B**) Expression of the recombinant His-GK at different temperatures and analysis of the proteins by 10% SDS-PAGE. Lane 1 - Marker; Lane 2 - Pellet (16°C); Lane 3 - Supernatant (16°C); Lane 4 - Pellet (20°C); Lane 5 - Supernatant (20°C); Lane 6 - Pellet (24°C); Lane 7 - Supernatant (24°C); Lane 8 - Pellet (37°C); Lane 9 - Supernatant (37°C). (**C**) Effect of induction time periods on the His-GK expression. His-GK was induced with 0.5 IPTG at 16°C at varying intervals of time, and the proteins were analysed by 10% SDS-PAGE. Lane 1 - Marker; Lane 2 - Pellet (2 h); Lane 3 - Supernatant (2 h); Lane 4 - Pellet (4 h); Lane 5 - Supernatant (4 h); Lane 6 - Pellet (6 h); Lane 7 - Supernatant (6 h); Lane 8 - Pellet (16 h); Lane 9 - Supernatant (16 h).

The coding sequence of GK was cloned into the pET24-a(+) vector and the construct was transformed into *E. coli* (BL21) and overexpressed. However, analysis of the protein fractions by10% SDS-PAGE, revealed that His-GK was expressed as IBs with a molecular weight of ∼55 kDa (Figure 2A).

To bring the protein into a soluble form, various parameters were trialled. First, different concentrations of IPTG (0.2, 0.5 and 1.0 mM) were used to determine the optimal concentration of IPTG for the expression of His-GK in *E. coli* at 37°C. However, the target protein was expressed as IBs even at 0.2 mM IPTG, although less protein was expressed at this concentration of IPTG compared with that at 0.5 mM and 1.0 mM IPTG (Figure 2A). Temperature is an important parameter that affects the expression of recombinant proteins. Therefore, the expression of His-GK was induced at different temperatures (16, 20, 24 and 37°C). However, temperature did not affect the solubility of His-GK; even at low temperature (16°C) the proteins were expressed as IBs (Figure 2B). Various induction time periods (2, 4, 6 and 16 h) were also examined and had no effect on His-GK solubility (Figure 2C).

**Figure 3:**
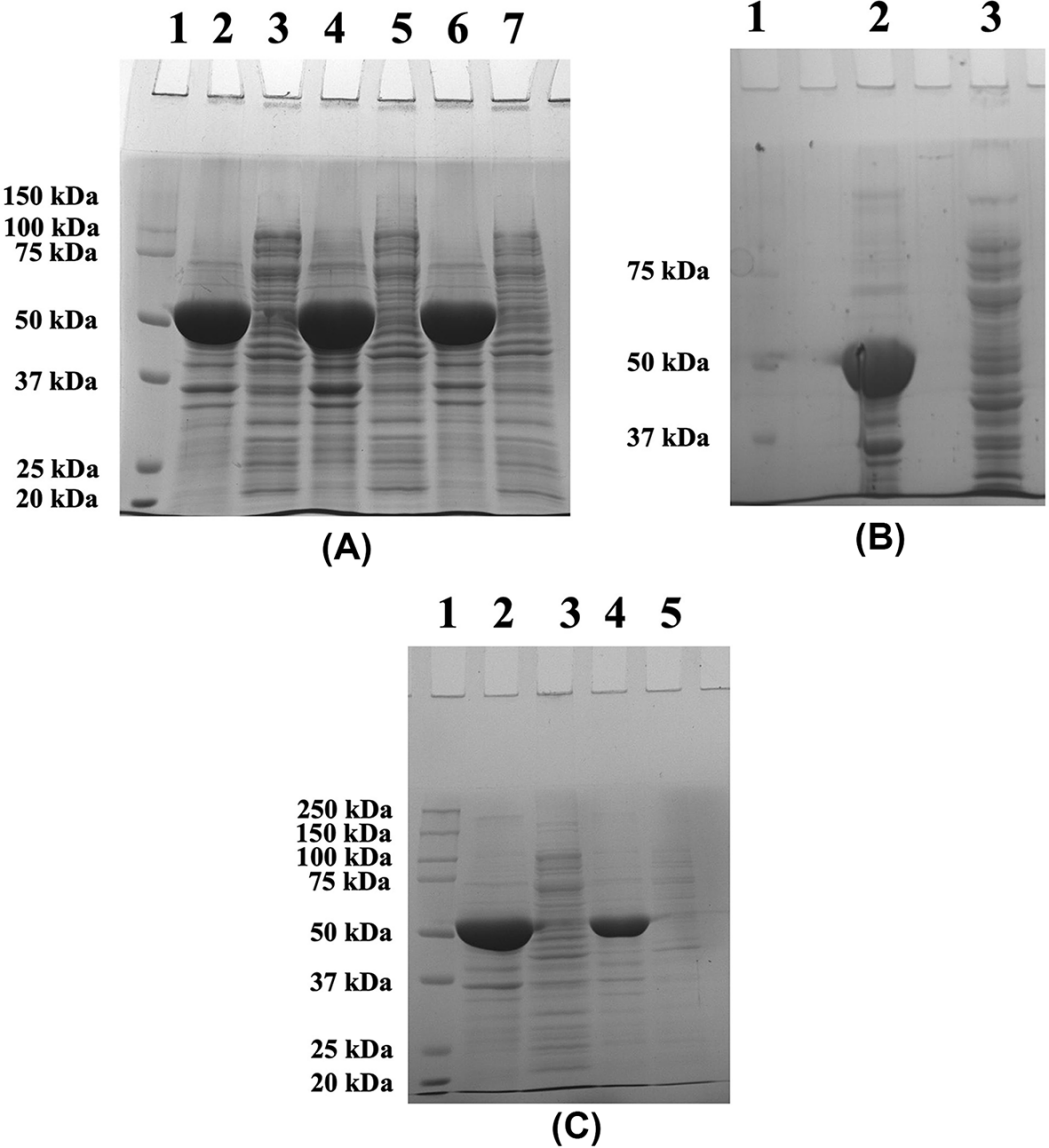
Effect of culture media and additives on His-GK solubility (**A**) The culture was grown in TB media with different concentrations of glycylglycine (Gly-Gly) and the proteins were analysed by 10% SDS-PAGE. Lane 1 - Marker; Lane 2 - Pellet (No Gly-Gly); Lane 3 - Supernatant (No Gly-Gly); Lane 4 - Pellet (10 mM Gly-Gly); Lane 5 - Supernatant (10 mM Gly-Gly); Lane 6 - Pellet (500 mM Gly-Gly); Lane 7 - Supernatant (500 mM Gly-Gly). (**B**) Analysis of His-GK expression in auto-induction media with 1% glycerol. Lane 1 - Marker; Lane 2 - Pellet (1% glycerol); Lane 3 - Supernatant (1% glycerol). (**C**) Analysis of His-GK expression in the presence of various concentrations of ethanol. Lane 1 - Marker; Lane 2 - Pellet (3% EtOH); Lane 3 - Supernatant (3% EtOH); Lane 4 - Pellet (5% EtOH); Lane 5 - Supernatant (5% EtOH).

Expression of recombinant proteins in different culture media reduces aggregation of recombinant proteins in *E. coli*; hence, the His-GK construct was expressed in various culture media and additives were also included in the media. A greater cell mass was produced with TB and auto-induction media (∼5.0 g cells per 1 L culture) as compared with LB (∼3.5 g cells per 1 L culture); however, the protein was expressed as IBs (Figures 3A,B). Consequently, LB medium was utilised in the following experiments. Glycylglycine (Figure 3A), glycerol (Figure 3B) and ethanol (Figure 3C) were also added to the culture media in individual experiments, but the protein was still found in IBs.

### Effect of solubilising agents on His-GK solubility from IBs

**Figure 4:**
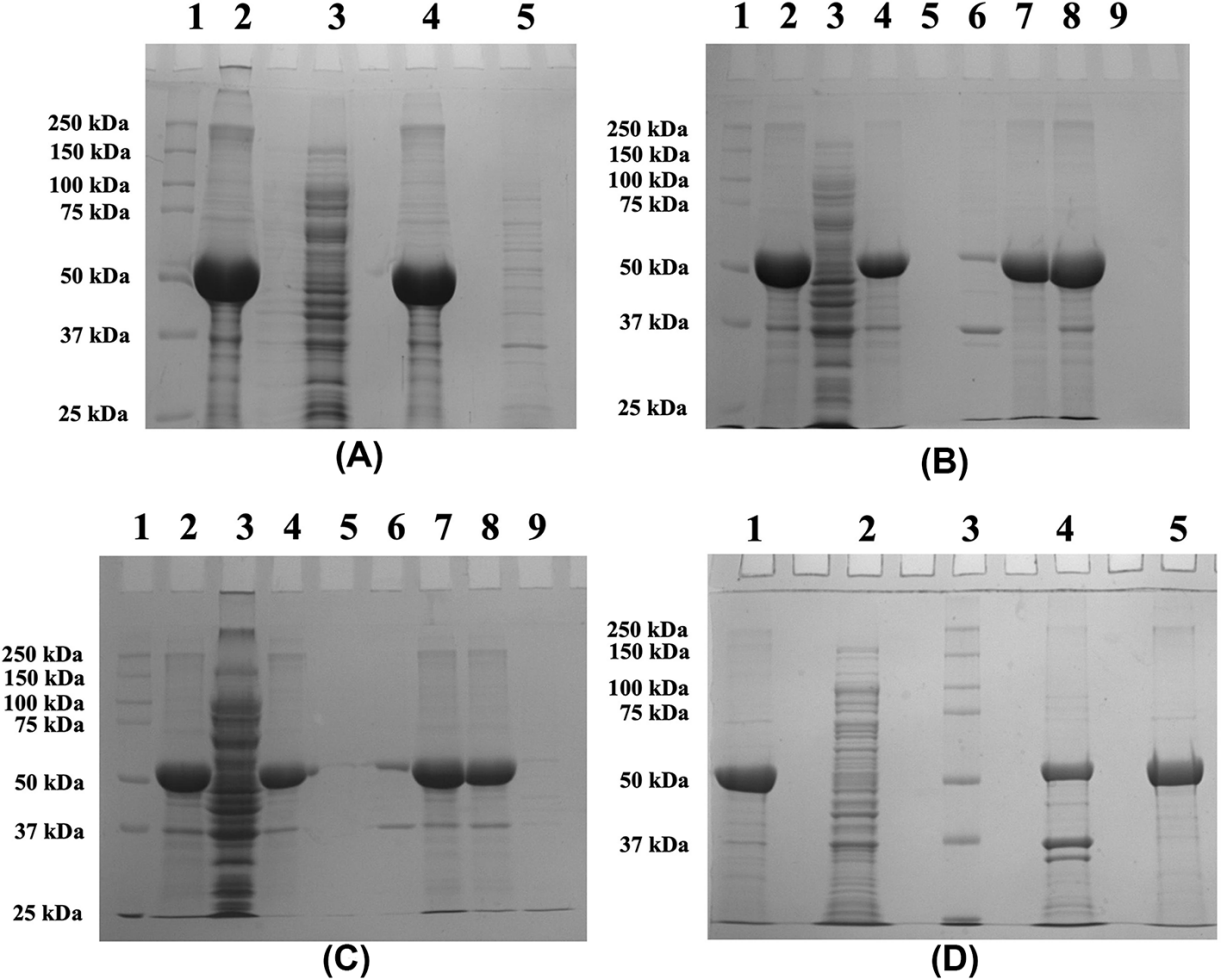
Effect of solubilizing agents on His-GK solubility (**A**) The IBs were dissolved in 1% CHAPS and the proteins were analysed by 10% SDS-PAGE. Lane 1 - Marker; Lane 2 - Pellet (control); Lane 3 - Supernatant (control); Lane 4 - Pellet (1% CHAPS); Lane 5 - Supernatant (1% CHAPS). (**B**) Arginine, CTAB and DMSO were used in separate experiments and the proteins were resolved by 10% SDS-PAGE. Lane 1 - Marker; Lane 2 - Original pellet; Lane 3 - Original supernatant; Lane 4 - Pellet (50 mM arginine); Lane 5 - Supernatant (50 mM arginine); Lane 6 - Pellet (0.5% CTAB); Lane 7 - Supernatant (0.5% CTAB); Lane 8 - Pellet (5% DMSO); Lane 9 - Supernatant (5% DMSO). (**C**) GdnHCl, SDS and urea were used to dissolve IBs and the proteins were resolved by 10% SDS-PAGE. Lane 1 - Marker; Lane 2 - Original pellet; Lane 3 - Original supernatant; Lane 4 - Pellet (2 M GdnHCl); Lane 5 - Supernatant (2 M GdnHCl); Lane 6 - Pellet (1% SDS); Lane 7 - Supernatant (1% SDS); Lane 8 - Pellet (2 M urea); Lane 9 - Supernatant (2 M urea). (**D**) 8 M urea was used to denature the protein and the fractions were analysed by 10% SDS-PAGE. Lane 1 - Original pellet; Lane 2 - Original supernatant; Lane 3 - Marker; Lane 4 - Pellet (8 M urea); Lane 5 - Supernatant (8 M urea).

**Figure 5:**
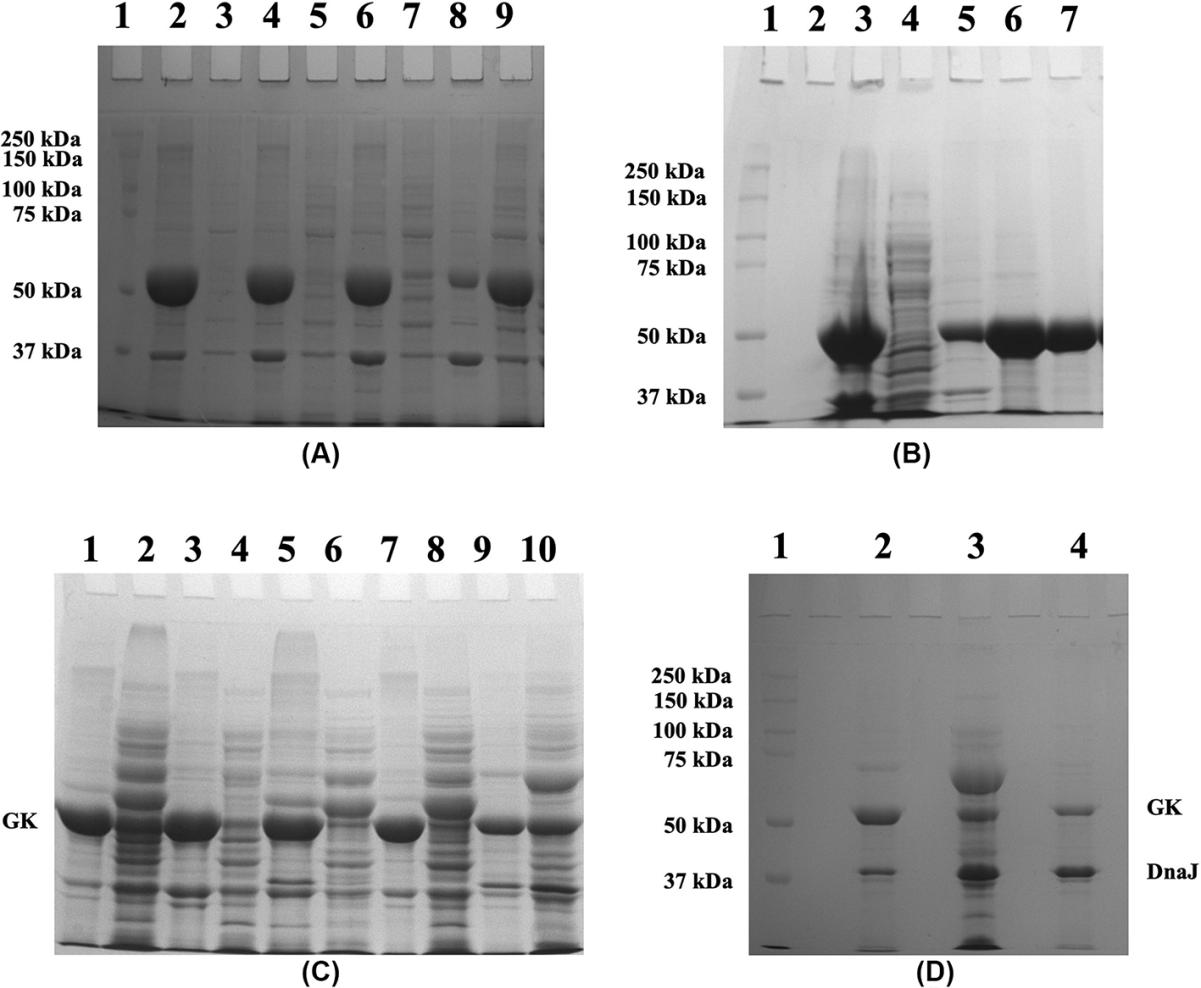
Purification of His-GK solubilized with sarkosyl and co-expressed with pKJE7 (**A**) The IBs were washed and resuspended in various concentrations of sarkosyl overnight at 4°C as described in Materials and methods, and the proteins resolved by 10% SDS-PAGE. Lane 1 - Marker; Lane 2 - Pellet (no sarkosyl); Lane 3 - Supernatant (no sarkosyl); Lane 4 - Pellet (0.1% Sarkosyl); Lane 5 - Supernatant (0.1% Sarkosyl); Lane 6 - Pellet (0.2% Sarkosyl); Lane 7 - Supernatant (0.2% Sarkosyl); Lane 8 - Pellet (1% Sarkosyl); Lane 9 - Supernatant (1% Sarkosyl). (**B**) The supernatant, after sarkosyl (1%) treatment, was purified using HisTrap HP column and the proteins were analysed by 10% SDS-PAGE. Lane 1 - Marker; Lane 3 - Pellet (control); Lane 4 - Supernatant (control); Lane 5 - Pellet (1% sarkosyl); Lane 6 - Supernatant (1% sarkosyl); Lane 7 - Purified His-GK. (**C**) His-GK was co-expressed with chaperone plasmids and the proteins were analysed by 10% SDS-PAGE. Lane 1 - Pellet (pG-Tf2); Lane 2 - Supernatant (pG-Tf2); Lane 3 - Pellet (pTf16); Lane 4 - Supernatant (pTf16); Lane 5 - Pellet (pG-KJE8); Lane 6 - Supernatant (pG-KJE8); Lane 7 - Pellet (pGro7); Lane 8 - Supernatant (pGro7); Lane 9 - Pellet (pKJE7); Lane 10 - Supernatant (pKJE7). (**D**) Purification of His-GK co-expressed with pKJE7 and analysis of the proteins by 10% SDS-PAGE. Lane 1 - Marker; Lane 2 - Pellet; Lane 3 - Supernatant; Lane 4 - Purified His-GK.

Since none of the *in vivo* methods resulted in expression of His-GK in a soluble form in *E. coli*, several solubilising agents were used to dissolve the IBs *in vitro*. Zwitterionic detergent CHAPS (1%), arginine (50 mM) and DMSO (5%), added individually, did not improve solubility of His-GK and the IBs (Figure 4A,B). However, addition of CTAB (0.5%) to the buffer containing isolated IBs did solubilised the IBs (∼90%) (Figure 4B), however, the protein was found to be in inactive form. The IBs were then dissolved in a solubilising buffer containing different denaturants such as GdnHCl (2 M), urea (2 M) and ionic denaturant SDS (1%). No improvement in protein solubility was observed with GdnHCl and urea, whereas 1% SDS significantly improved the solubility of IBs (Figure 4C), but the protein was in an inactive form. A high concentration of urea (8 M) could also solubilise the IBs (≥60%) (Figure 4D), but the protein was again inactive, even after refolding by stepwise dialysis method. The IBs were effectively dissolved in an anionic detergent (1% sarkosyl) (Figure 5A) and the recombinant protein was found to be active in the presence of sarkosyl. Therefore, the proteins were purified (Figure 5B) according to ‘Purification of recombinant His-GK’ in the Materials and methods section; however, the protein was inactive.

### Effect of molecular chaperones on His-GK solubility

His-GK was co-expressed with different molecular chaperones to solubilise the recombinant protein. However, there was no significant protein solubility in the case of the chaperone plasmids such as pG-KJE8, pGro7, pG-Tf2 and pTf16 (Figure 5C, lanes 1–8). Comparatively, a significant increase in the solubility of His-GK (∼50%) was observed with pKJE7 (Figure 5C; lanes 9–10). Furthermore, to optimize the soluble expression of His-GK, various strategies were employed such as simultaneous induction of the chaperones and the target protein and induction of chaperones before IPTG induction. Induction of the chaperones before IPTG induction showed a relatively better result in protein solubility compared with simultaneous induction (Supplementary Figure S1).

### Purification and characterisation of His-GK including determination of Michaelis–Menten constants

His-GK obtained from a 1-L culture by co-expressing with pKJE7 plasmid was purified using a HisTrap HP column. An outline of the different stages of purification is presented in Table 1. The eluted protein was further purified using a desalting column followed by an SEC column, and the purification of His-GK was confirmed by 10% SDS-PAGE analysis and chromatograms (Figure 5D; Supplementary Figure S2). Purified His-GK was then characterised following enzyme kinetics. The optimal parameters for the enzyme were calculated and presented are in Table 2. In addition, the Kmapp for the substrate and co-factors of human His-GK were determined (Table 3).

**Table 1:** Outline of purification of human His-GK (from 1 L culture) co-expressed with molecular chaperone (pKJE7) in *E. coli*

Stages of purification	Protein (mg)	Total units^*^ (U)	Specific activity (U/mg of protein)	Yield (%)	Folds of purification
Pellet	66.50	–	–	–	–
Supernatant	227.16	59.20	0.261	–	–
HisTrap HP column	102.86	42.40	0.412	71.62	157.94
HiTrap desalting column	90.01	34.60	0.384	58.45	147.28
HiPrep™ 16/60 Sephacryl™ S-200 HR	30.01	23.10	0.770	39.02	294.92

* One unit of His-GK activity is the amount of enzyme that catalyzes one µmole of NADH oxidation per min under standard reaction conditions as described in Materials and methods.

**Table 2:** Effect of the various factors on the purified human His-GK activity

Factors	Human His-GK (mean ± SEM)
Buffers (50 mM, pH 7.5)	
TEA	0.486 ± 0.054
Imidazole	0.468 ± 0.060
Tris-HCl	0.438 ± 0.060
PBS	0.300 ± 0.006
HEPES	0.342 ± 0.042
Metal ions (40 mM)	
KCl	0.660 ± 0.018
LiCl	0.165 ± 0.036
NaCl	0.290 ± 0.04
MgCl_2_	0.0
CaCl_2_	0.0

Specific activity for human His-GK was calculated under standard reaction conditions as described in Materials and methods. Specific activities are given as mean ± SEM values from four independent experiments (*n*=4).

**Table 3:** Apparent Michaelis-Menten constants (*K*m app) for the substrate and co-factors of human His-GK

Substrate or co-factors	*K*mapp for human His-GK
PEP	0.223 mM
Glycerol	5.022 µM
ATP	0.767 mM

Lineweaver–Burk plots and Eadie-Hofstee plots were used for the determination of *K*m app of human His-GK.

**Figure 6:**
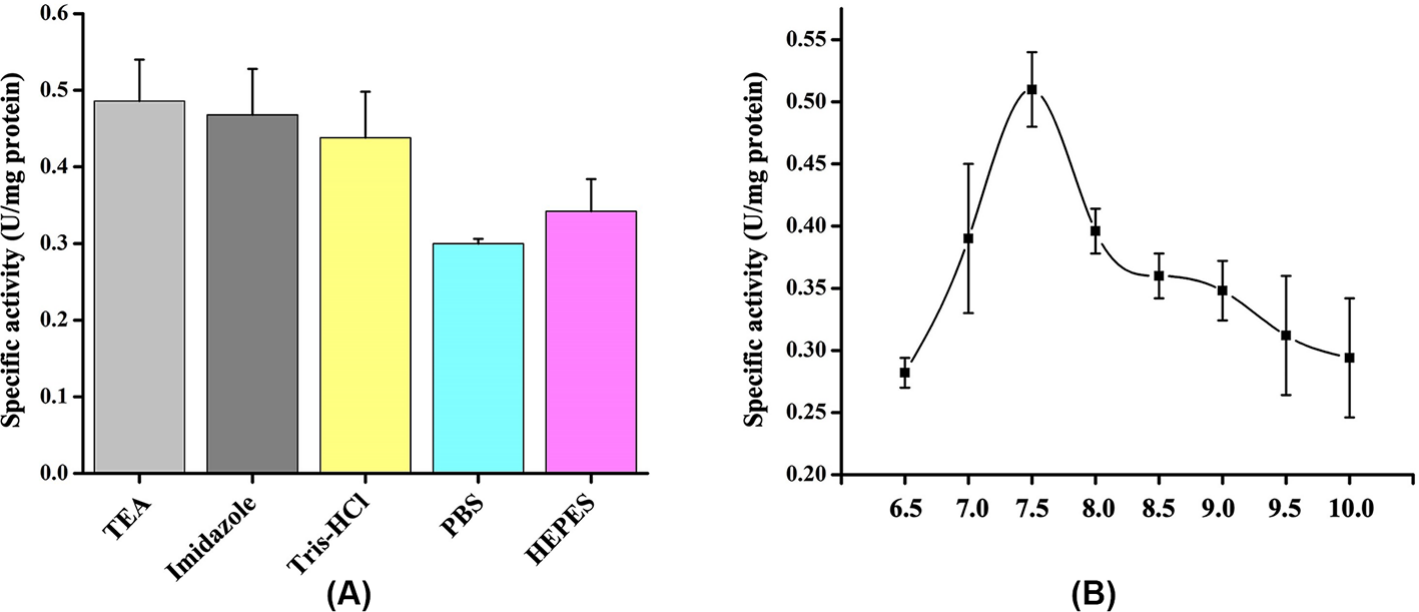
Effect of buffers and pH on His-GK activity The purified enzyme from the SEC column was used for enzyme kinetics as described in Materials and methods. (**A**) Effect of various buffers (50 mM) on the specific activity of His-GK. (**B**) Effect of pH (6.5–10.0) on the specific activity of His-GK using TEA buffer.

The molecular weight of His-GK from native-PAGE analysis was found to be ∼100 kDa (Supplementary Figure S3) in comparison with ∼55 kDa as determined by 10% SDS-PAGE (Figure 2A). This indicated that His-GK existed as a dimer in its bioactive form. Several buffers (TEA, imidazole, Tris-HCl, PBS, and HEPES) were tested to identify the optimal buffer for His-GK activity. Optimal enzyme activity was observed in 50 mM TEA buffer (Figure 6A), although His-GK activity in imidazole and Tris-HCl buffers was very similar to that of TEA buffer. The optimal pH for His-GK activity was determined to be pH 7.5 (Figure 6B). A sharp rise in His-GK activity was evident as the pH increased from 6.5 to 7.5, but a gradual decrease in activity was observed above pH 7.5. However, His-GK activity was detected from pH 7.0 to pH 8.0.

**Figure 7:**
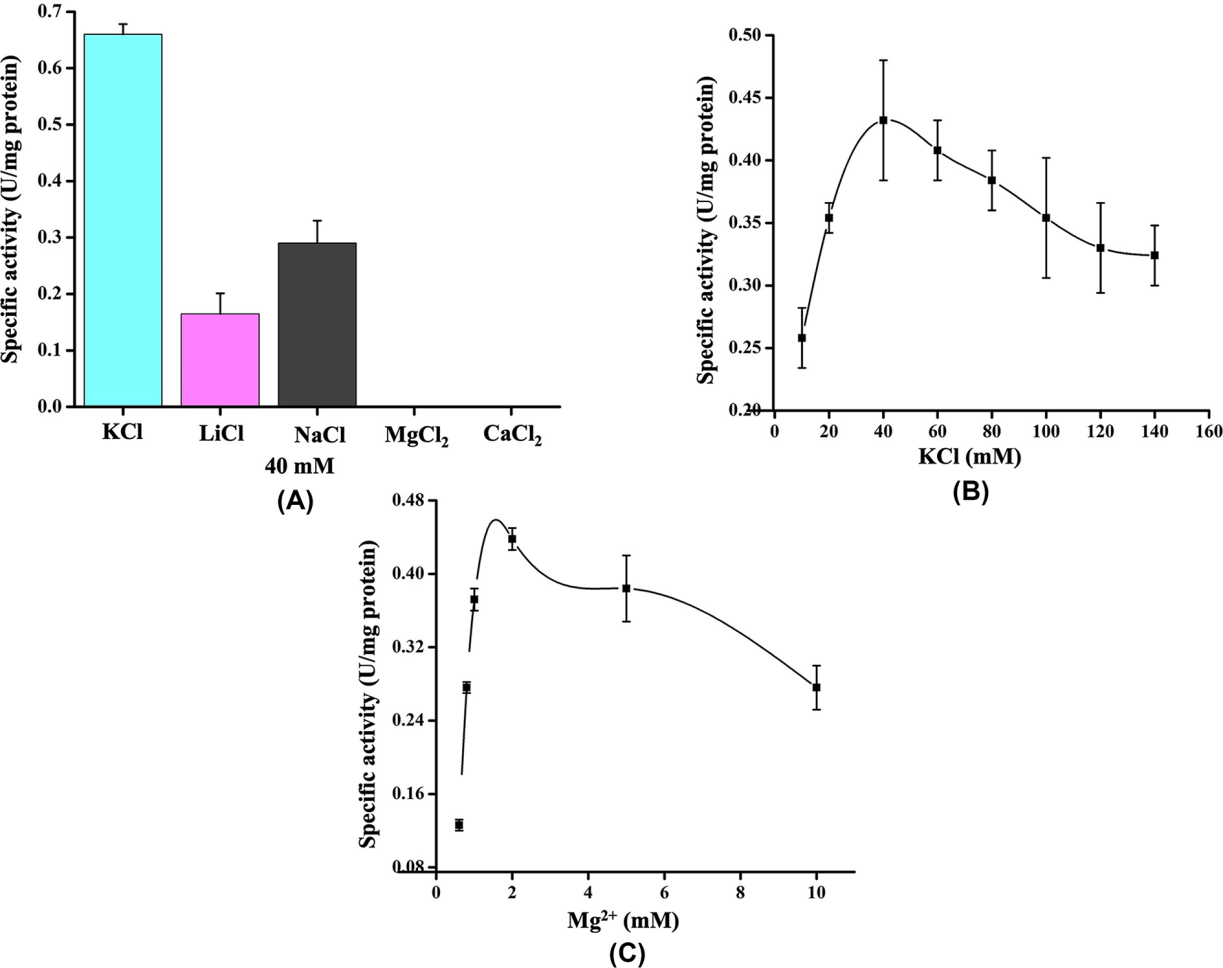
Effect of metal ions on His-GK activity The ideal metal ion for His-GK activity was also determined as described in Material and Methods. (**A**) Effect of various metal ions (KCl, LiCl, NaCl, MgCl_2_ and CaCl_2_) on the specific activity of His-GK. (**B**) Effect of various concentrations of KCl (10–140 mM) on the specific activity of His-GK. (**C**) Effect of different concentrations of Mg^2+^ (0.6–10 mM) on the specific activity of His-GK.

Investigation of the effect of some metal ions (CaCl_2_, KCl, LiCl, MgCl_2_ and NaCl) on the activity of His-GK revealed that KCl was the optimal cation. Replacement of KCl with MgCl_2_ or CaCl_2_ did not yield His-GK activity, whereas very low His-GK activity was observed with NaCl and LiCl (Figure 7A). To determine the optimal concentration of KCl for His-GK activity, a wide range of KCl concentration (10, 20, 40, 60, 80, 100, 120 and 140 mM) were tested. The optimal concentration of KCl was 40 mM, and a decrease in His-GK activity was observed when the concentration of KCl was greater than 40 mM (Figure 7B). ATP binds to the Mg^2+^ ion; thus, the Mg^2+^ ion is necessary for GK activity. Therefore, along with the optimal concentration of KCl (40 mM), different concentrations of MgSO_4_ (0.6, 0.8, 1.0, 2.0, 5.0 and 10.0 mM) were tested and 2.0 mM was found to be the optimal concentration of Mg^2+^ for His-GK activity. A gradual decrease in GK activity was observed as the concentration of Mg^2+^ exceeded 2.0 mM (Figure 7C).

**Figure 8:**
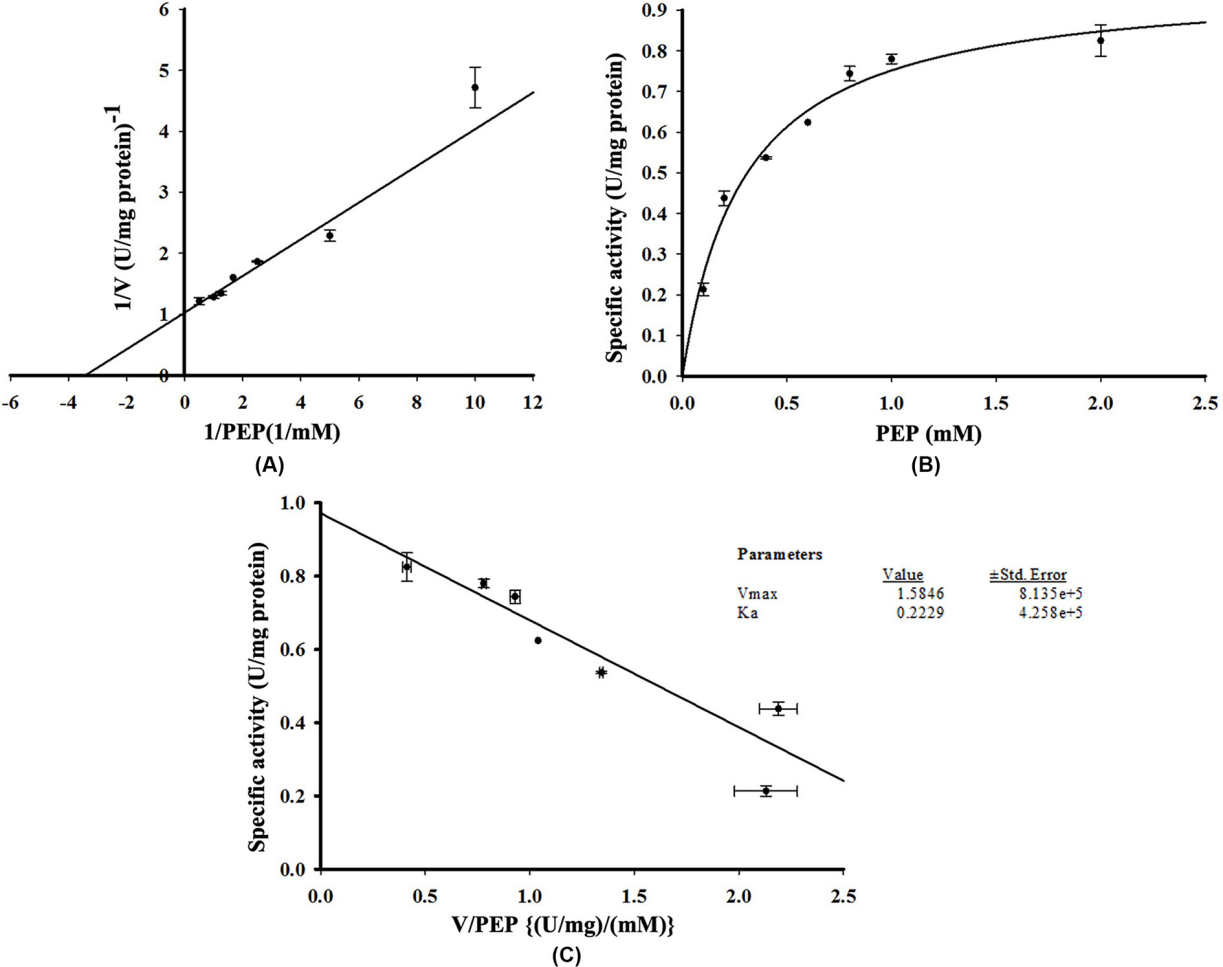
Effect of different concentrations of PEP on His-GK activity Different concentrations of PEP (0.1–2.0 mM) were used to determine the *V*max and *K*m of His-GK for PEP. (**A**) Lineweaver-Burk Plot showing Vmax and Km of PEP for His-GK activity. (**B**) Michaelis-Menten plot of His-GK in presence of various concentrations of PEP. (**C**) Eadie–Hofstee plot for PEP with *V*max=1.585 U/mg protein and *K*m=0.223 mM (R^2^=0.967).

To determine the effect of PEP on His-GK activity, several concentrations of PEP (0.1, 0.2, 0.4, 0.6, 0.8, 1.0 and 2.0 mM) were used and a hyperbolic curve, obeying Michaelis–Menten kinetics, was observed. Using SigmaPlot 14.5, the Vmax of PEP for His-GK activity was 1.585 U/mg protein and *K*m was calculated to be 0.223 mM with a positive correlation (*R*^2^=0.967). Lineweaver–Burk (Figure 8A), Michaelis–Menten (Figure 8B) and Eadie–Hofstee graphs (Figure 8C) were plotted using SigmaPlot 14.5.

**Figure 9:**
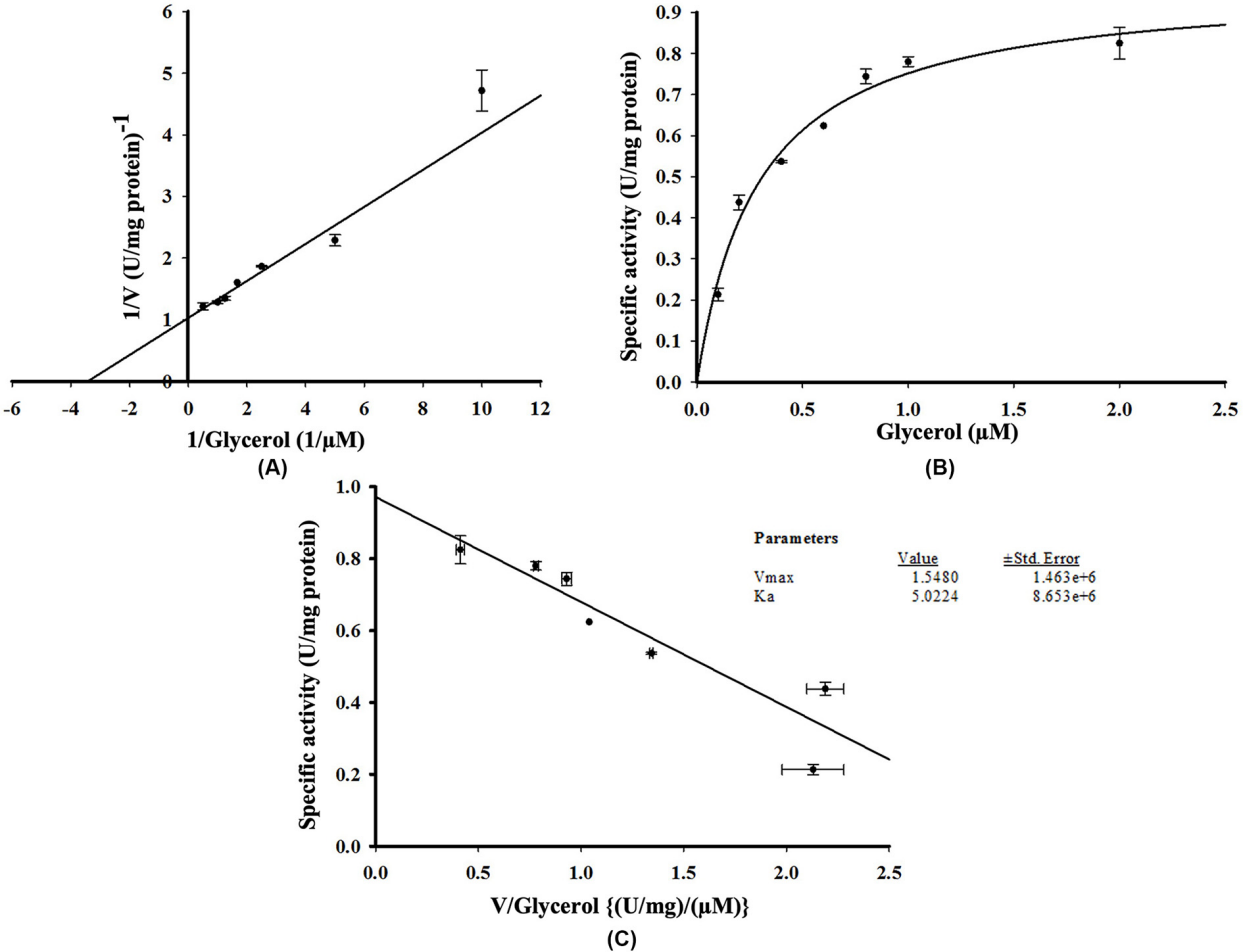
Effect of different concentrations of glycerol on His-GK activity Different concentrations of glycerol (1–50 µM) were used to determine the *V*max and *K*m of His-GK for glycerol. (**A**) Lineweaver–Burk plot showing *V*max and *K*m of glycerol for His-GK activity. (**B**) Michaelis–Menten plot of His-GK in presence of various concentrations of glycerol. (**C**) Eadie–Hofstee plot for glycerol in His-GK with *V*max = 1.548 U/mg protein and *K*m = 5.022 µM (*R*^2^ = 0.927).

**Figure 10:**
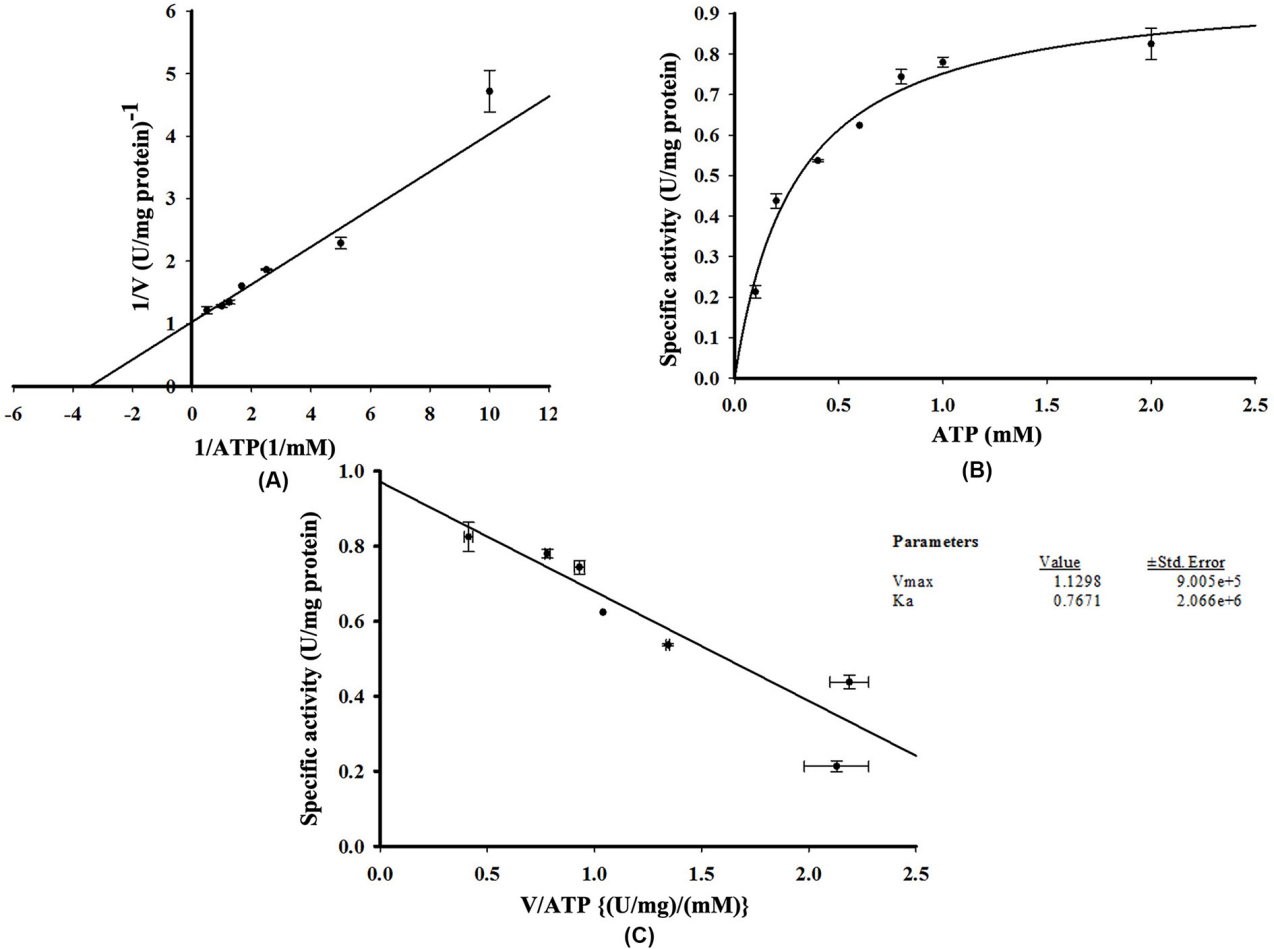
Effect of different concentrations of ATP on His-GK activity Different concentrations of ATP (0.2 to 2.0 mM) were used to determine the *V*max and *K*m of His-GK for ATP (**A**) Lineweaver–Burk plot showing *V*max and *K*m of ATP for His-GK activity. (**B**) Michaelis–Menten plot of His-GK in presence of various concentrations of ATP. (**C**) Eadie–Hofstee plot for ATP with *V*max=1.130 U/mg protein and *K*m=0.767 mM (*R*^2^=0.928).

To determine the *K*m of His-GK for its substrate and co-factors, various concentrations of glycerol (1, 2, 4, 6, 8, 10, 20 and 50 µM) and ATP (0.2, 0.4, 0.6, 0.8, 1.0 and 2.0 mM) were used. The *V*max and *K*m of His-GK with glycerol were 1.548 U/mg protein and 5.022 µM, respectively, with a positive correlation (*R*^2^=0.927). Lineweaver–Burk (Figure 9A), Michaelis–Menten (Figure 9B) and Eadie–Hofstee graphs (Figure 9C) were also plotted for the substrate. For ATP, as observed from the Lineweaver–Burk plot (Figure 10A), the *V*max was 1.130 U/mg protein and *K*m was calculated to be 0.767 mM with a positive correlation (*R*^2^=0.928). Michaelis–Menten (Figure 10B) and Eadie–Hofstee graphs (Figure 10C) were also plotted. In the present study, the specific activity of His-GK was approximately 0.780 U/mg protein. Comparison of various parameters of recombinant human His-GK with those of GK from different organisms is shown in Table 4.

**Table 4:** Comparative analyses of different parameters for the enzyme glycerol kinase from different organisms including human His-GK

Sl. No	Organism	Specific activity (U/mg protein)	Subunit mol. wt. (kDa)	pI	pH	*K*m for glycerol (mM)	*V*max for glycerol (U/mg protein)	*K*m for ATP (mM)	*V*max for ATP (U/mg protein)	Optimised values for co-factors	Inhibitors	Reference
1.	Human (His-GK)	0.780	∼55.0		7.5	0.005	1.548	0.767	1.130	KCl (40 mM), MgSO_4_ (2 mM), PEP (2 mM); * K*m for PEP (0.223 mM), *V*max (1.585 U/mg)		Present study
2.	Human (adrenal homogenate)	0.216	57.5			0.003	0.283 ± 0.007	0.022	0.354 ± 0.007		ADP, AMP	[57]
3.	Human leukocytes					0.042	1.40 × 10^−5^	0.394	3.00 × 10^−5^			[56]
4.	Human kidney					0.034	5.60 × 10^−5^	0.412	6.30 × 10^−5^			[56]
5.	Human (fibroblast)		57.5		7.0	0.004	0.52 × 10^−5^	0.075	0.60 × 10^−5^		ADP, AMP	[56]
6.	Human (liver)		57.5			0.042	3.10 × 10^−5^	0.317	3.70 × 10^−5^		ADP, AMP, ethanol	[56]
7.	Rat liver	0.190	61.0		9.0	0.035		0.035			ADP, AMP, ethanol	[51]
8.	Beef liver	0.055	60.6		7.5	0.020		0.010			ADP, AMP, ethanol	[51]
9.	Human liver	0.053			7.5	0.002		0.010				[51]
10.	*T. brucei gambiense*	2.900	56.4		7.0	4.360	3.10				ADP	[46]
11.	*T. brucei*	173.700 ± 6.1	53.0		8.6	0.169 ± 0.02	206.6 ± 21.8	0.246 ± 0.019	203.7 ±18.8		ADP	[61]
12.	*E. coli*	32.000	57.0		7.0-9.2	0.010		0.100 mM			Fructose-1,6 diphosphate, IIA^Glc^	[60]
13.	*Candida mycoderma*		62.0		7.5	0.035		0.055			AMP	[51]
14.	*T. brucei*	4.410 × 10^4^	53.0		7.4	0.260±0.02		0.190 ± 0.02			ADP, 1,2-propanediol	[19]
15.	*T. brucei*	4.410 × 10^4^	53.0		9.5	0.170± 0.03		0.260 ± 0.02			ADP, 1,2-propanediol	[19]
16.	*E. coli*				7.5	0.001					Fructose-1,6- diphosphate	[63]
17.	*Salmonella typhimurium*				9.0	0.007					III^glc^, FDP	[58]
18.	*Clostridium beijerinckii*		55.5		9.5	0.356 ± 0.027		0.135 ± 0.016 mM				[62]
19.	*Thermococcus kodakarensis*		55.9		8.5	0.111 ± 0.00		0.015 ± 0.001				[44]
20.	*T. brucei*	133.000	56.4	8.6	8.0	0.44 ± 0.09	226 ± 56	0.240 ± 0.09	183 ± 20			[52]
21.	*E. coli*	3.200	2.8 × 10^5^		7.5- 9.5	0.001	100	4.000			FDP	[2]

## Discussion

Overexpression of recombinant protein in *E. coli* often results in aggregation of the expressed protein into IBs [21]. This is owing to the lack of advanced molecular apparatuses for post-translational modifications in *E. coli*, which results in formation of IBs [22]. The occurrence of IBs sets an obstacle in the production and purification of recombinant proteins in *E. coli* [23]. Optimisation of various parameters such as inducer concentrations, induction time periods, temperatures, media and different additives can partially help in overcoming the formation of IBs. IPTG concentration needs to be optimised for solubilising the recombinant protein while being expressed in *E. coli* [24]. Temperature is an important parameter for optimising the solubility of a protein [25,26] and protein solubility decreases with increasing in temperature and is indicative of protein denaturation [27]. However, despite expressing His-GK at 16°C, the protein was still found in IBs. A short induction period is also reported to play a role in preventing protein aggregation [28], but in the present study, His-GK expression was not affected by different induction time intervals. But in most cases, in the present study too, modifying the culture conditions do not improve protein solubility. and the protein. Expression and solubility of recombinant proteins is affected by the composition and richness of culture media [29]. *E. coli* carrying the His-GK construct was grown in various culture media and a reduced cell mass was produced in LB compared with TB and auto-induction media; however, there was no effect on protein solubility. A previous study showed that the solubility of mycobacterial proteins was enhanced by adding glycylglycine to the culture media [30]. In addition, the presence of glycerol in the culture media is reported to provide structural and functional stability to an expressed protein; thereby, increasing the solubility of the protein [31]. Meanwhile, a study on human pro-insulin demonstrated that the addition of a low concentration of ethanol in the culture media helped to decrease protein aggregation [32]. In this study, individual addition of glycylglycine, 1% glycerol, and 3% ethanol to the culture media did not facilitate solubilisation of His-GK. Hence, various solubilising agents were explored to solubilise the protein *in vitro*.

IBs can also be solubilised using high concentrations of chaotropic agents and then refolded to their native state through various refolding procedures [33]. However, the solubilisation of IBs utilizing mild solubilisation conditions protects the native structure of the proteins, diminishes protein aggregation, and recovers bioactive proteins from IBs [34]. Solubilising agents such as CHAPS, arginine, CTAB, DMSO, GdnHCl, urea, SDS and sarkosyl are used to solubilise the recombinant proteins [33,35,36]. In the present study, various solubilising agents (1% SDS, 0.5% CTAB, 8 M urea and 1% sarkosyl) were successful in solubilising the IBs. However, the His-GK obtained after treatment with the above-mentioned solubilising agents was inactive, even after refolding (in the case of 8 M urea). Total unfolding of the solubilized protein increases the chance of aggregation during refolding, which subsequently leads to low recovery of bioactive protein [33]. The denaturation can be irreversible in instances where the unfolded protein is susceptible to aggregation [37], which might be the reason for obtaining inactive His-GK in the present study. The breakage of disulphide bonds may be the barriers for refolding of an unfolded protein [38].

In our quest to produce bioactive soluble protein, a coordinated network of several molecular chaperones is employed to decrease aggregation of target proteins in *E. coli* systems [39–41]. Therefore, to improve the solubility of His-GK, a set of five chaperones (pG-KJE8, pGro7, pG-Tf2, pKJE7 and pTf16) were evaluated in this study. Plasmid pKJE7, consisting of chaperone sets dnaK-dnaJ-grpE, resulted in the highest proportion of soluble His-GK protein when co-expressed with His-GK. DnaK-dnaJ-grpE is a member of the Hsp 70 family possessing isomerase activity to assist the folding of multidomain proteins by reversing and inhibiting intramolecular misfolding [39]. DnaK/DnaJ interacts with the hydrophobic segments of the unfolded polypeptide to maintain solubility; while GrpE facilitates the dissociation of DnaK and DnaJ from the polypeptide [42,43]. The recombinant human His-GK protein, obtained through co-expressing with pKJE7, was purified, and the molecular weight of the human His-GK monomer was ∼55 kDa. This was congruent with other studies, which reported that the molecular weight of the GK monomer is in the range of 50–60 kDa [44–46].

Various parameters were explored to optimise the activity of purified human His-GK. TEA buffer (50 mM) at pH 7.5 was the ideal buffer for human His-GK activity. TEA buffer is the most commonly used buffer for GK enzyme assays [19,47]; however, buffers such as sodium phosphate [48] and Tris-HCl [49,50] are also used for GK enzyme assay.

His-GK activity was increased as the pH was increased from 6.0 to 7.5, indicating that human His-GK is pH sensitive. The pH for GK activity in other organisms is reported within a wide range, from pH 7.0 to pH 9.0 [44] but GK activity from human liver and cow liver are recorded to be active at pH 7.5, while that from rat liver is active at pH 9.0 [51]. *Trypanosoma brucei* GK activity is highest at pH 8.0 and decreases steadily beyond the pH range of 7.5–8.5 [52] while the maximal activity of GK from diapause eggs of the silkworm, *Bombyx mori*, is recorded at pH 8.5–9.0 [49]. However, GK from blood parasites, human African trypanosomes, is maximally active at pH 6.8 [53].

GK activity is regulated by co-factor like K^+^ [54]. KCl was revealed as the most suitable monovalent cation for His-GK activity in the present study. This is consistent with Krakow and Wang [19] who reported the importance of K^+^ as a co-factor in GK activity. KCl was used by Montell et al. [55] to check GK activity in human muscle cells, and in other human tissues, such as the liver and adrenal, KCl is a vital component for tissue homogenate preparation [56,57]. In contrast, Mg^2+^ is the preferred metal ion for *E. coli* GK activity and Mn^2+^ can replace it [2]. GK uses ATP as a phospho-donor; hence, Mg^2+^ is required for the catalytic activity [58]. In this study, 2 mM of Mg^2+^ was found to be optimal for 2 mM ATP substrate. Increasing the molar ratio of Mg^2+^ to ATP beyond the optimum value is reported to inhibit GK activity [50].

The interaction of His-GK with PEP, the intermediate substrate in the GK kinetics coupling reaction, was characterised and the optimal concentration of PEP for His-GK activity was 2 mM, in agreement with that reported by Krakow and Wang [19]. In the present study, the *V*max and *K*m of His-GK for PEP were 1.585 U/mg protein and 0.223 mM, respectively. The *V*max of His-GK for glycerol and ATP was approximately 1.548 U/mg protein and 1.130 U/mg protein, respectively, whereas the Km of His-GK for glycerol and ATP was calculated as 5.022 µM and 0.767 mM, respectively. A previous study reported that the *V*max value and *K*mapp of GK from human adrenal homogenate were 283.4 mM/min and 2.8 µM, respectively, for glycerol and 354.2 mM/min and 22.0 µM, respectively, for ATP [57]. *K*mapp values for glycerol and ATP from diapause eggs of the silkworm, *B. mori*, are found as 0.32 and 0.095 mM, respectively [49]. However, *V*max and *K*m recorded from GK of *T. brucei gambiense* were 3.10 μM/min and 4.36 mM, respectively [53]; while the Km value of GK from *Chaetomium thermophilum* for ATP was recorded as 0.052 mM [59].

The specific activity of GK from *E. coli* is reported to be 32 U/mg of protein [60], while that of GK from *T. brucei* is 173 U/mg of protein [61]. Seltzer et al. [62] reported that the specific activity of GK from human adrenal homogenate is 0.216 U/mg of protein. The GK enzyme activity of cultured human myotubes is reported to be quite low at 5.1 mU/mg protein [55]. In this study, the specific activity of human His-GK was found to be 0.780 U/mg protein.

In conclusion, this study indicated that molecular chaperones, specifically pKJE7, facilitated in expressing human His-GK in a soluble form for its subsequent purification and characterisation. This study also provided a basic background for the biochemical properties of human His-GK. GK is a promising drug target since it is present at the junction of carbohydrate- and lipid- metabolism; hence, further studies on this enzyme are essential to understand its possible role in T2DM.

## Supplementary Material

Supplementary Figures S1-S3

## Data Availability

Data related to this paper can be acquired on request.

## Abbreviations

ATP: adenosine 5′-triphosphate
BSA: bovine serum albumin
CHAPS: 3-[(3-cholamidopropyl) dimethylammonio]-1-propanesulfonate
CTAB: cetyltrimethylammonium bromide
DMSO: dimethyl sulfoxide
DTT: dithiothreitol
EDTA: ethylenediamine tetraacetic acid
G3P: sn-glycerol-3-phosphate
GdnHCl: guanidine hydrochloride
GKD: glycerol kinase deficiency
GSH: L-Glutathione reduced
GSSG: L-Glutathione oxidized
IBs: inclusion bodies
IPTG: isopropyl-beta-D-thiogalactopyranoside
Kmapp: apparent Michaelis-Menten constant
LB: Luria-Bertani
LDH: lactate dehydrogenase
NADH: nicotinamide adenine dinucleotide (reduced)
NCBI: National Center for Biotechnology Information
PDB: Protein Data Bank
PEP: phosphoenolpyruvate
PK: pyruvate kinase
PMSF: phenylmethylsulfonyl fluoride
SDS-PAGE: sodium dodecyl-sulfate-polyacrylamide gel electrophoresis
SEC: Size Exclusion Chromatography
T2DM: type 2 diabetes mellitus
TB: terrific broth
TEA: Triethanolamine
U: unit
