# Correction: Expression and characterisation of human glycerol kinase: the role of solubilising agents and molecular chaperones

**DOI:** 10.1042/BSR-2022-2258_COR

**Published:** 2023-10-20

**Keywords:** glycerol kinase, sarkosyl, solubilising agents, Type 2 diabetes mellitus

The authors of the original article “Expression and characterisation of human glycerol kinase: the role of solubilising agents and molecular chaperones” (doi: 10.1042/BSR20222258) would like to correct Figures 8-10.

**Figure 8:**
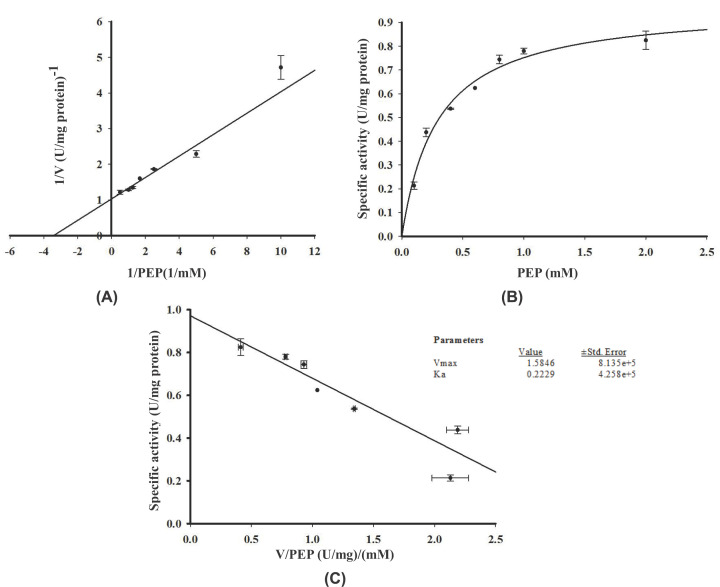
Effect of different concentrations of PEP on His-GK activity

After publication, readers identified that these figures presented the same data but with different label axes and different Vmax and Ka values. The Editorial Office identified that the figures and data provided in the articles original submission were correct, and that this error was introduced by the authors during the first revision. The authors state that this occurred due to copying and pasting the graphs when checking the alignment of the graphs in Figure 8, with that of Figures 9 and 10. The corrected figures appear below.

**Figure 9:**
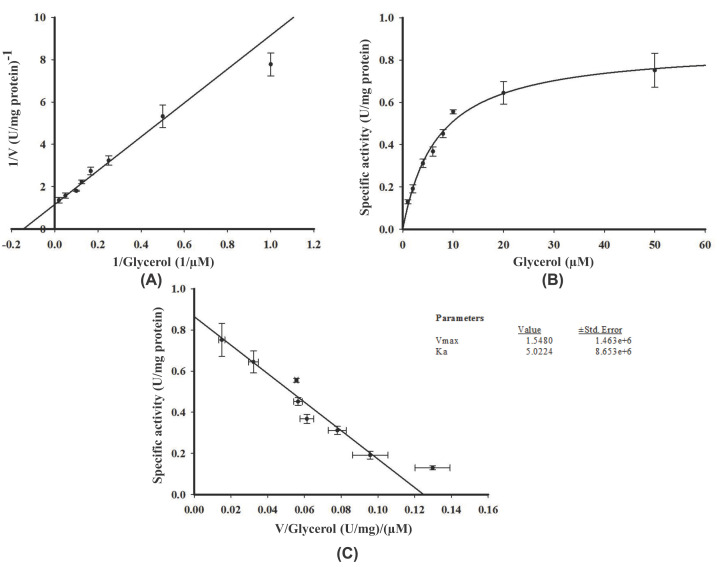
Effect of different concentrations of glycerol on His-GK activity

**Figure 10:**
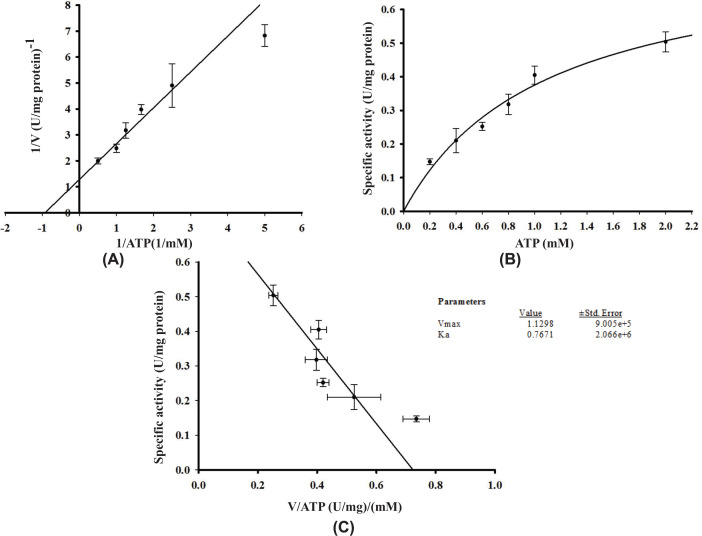
Effect of different concentrations of ATP on His-GK activity

The requested correction and related raw data have been assessed and agreed by the Editorial Board. The authors declare that these corrections do not change the results or conclusions of their paper.

