# Supplementary material for: Expression and characterisation of human glycerol kinase: the role of solubilising agents and molecular chaperones
Source: Biosci Rep. 2023 Apr 21;43(4):BSR20222258. doi: 10.1042/BSR20222258 (PMC10130975; doi:10.1042/BSR20222258)
Supplement: Supplementary Figures S1-S3 [file BSR-2022-2258_supp.pdf]

Supplementary Information

Expression and characterisation of human glycerol kinase: the role of solubilising agents and molecular chaperones

Riva Mary Rani, Superior Syngkli, Joplin Nongkhlaw and Bidyadhar Das*

Biological Chemistry Laboratory, Department of Zoology, North-Eastern Hill University, Shillong-793022, India

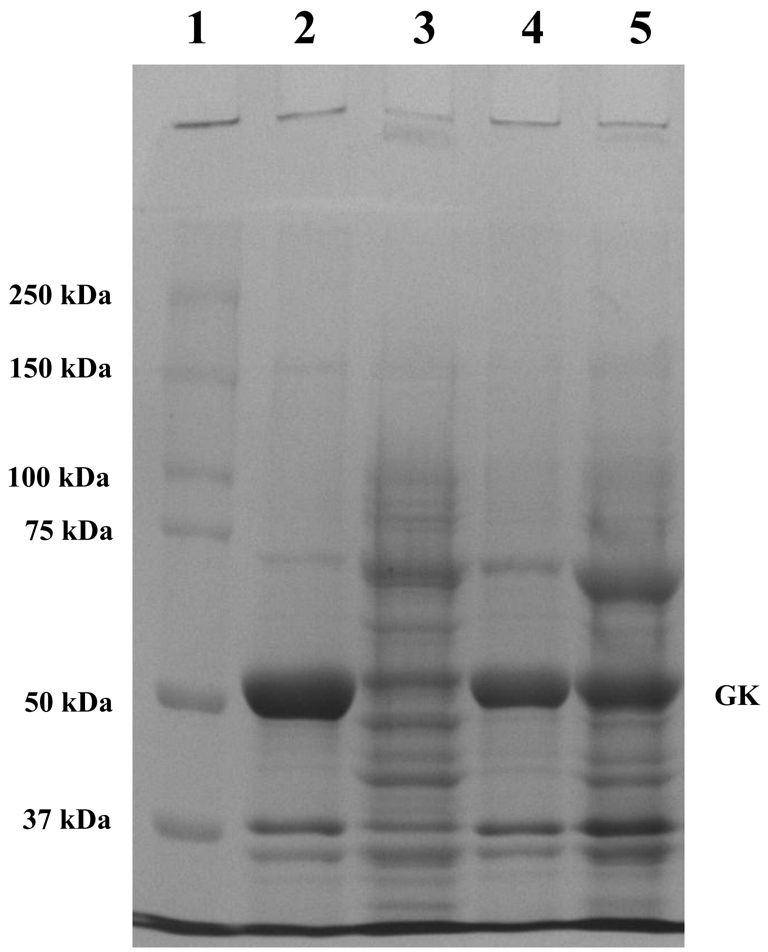

Figure S1. Effect of chaperone induction time on solubility of His-GK
Different chaperone induction time on solubility of His-GK was analyzed by 10% SDS-PAGE; in simultaneous induction, the chaperones as well as the target protein (His-GK) were induced at the same time; and in prior induction, the chaperones were induced after 2 h of culture and the target protein (His-GK) was induced 1 h after induction of chaperones. Lane 1 - Marker (1.0 μl); Lane 2 - Pellet (simultaneous induction); Lane 3 - Supernatant (simultaneous induction); Lane 4 - Pellet (prior induction); Lane 5 - Supernatant (prior induction).

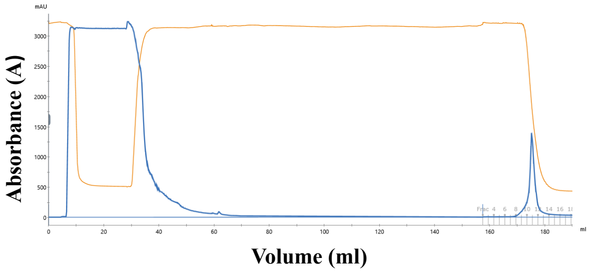

A

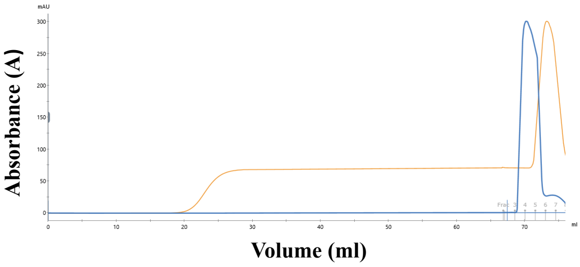

B

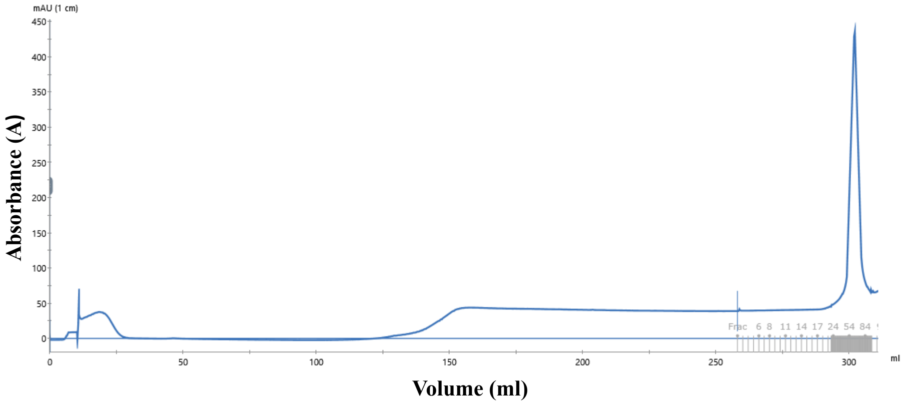

C

Figure S2. Chromatograms of purification of His-GK

The figure represents the chromatograms of purification of His-GK. The recombinant protein (His-GK) was co-expressed with the chaperones and purified as described in the "Materials and methods". **(A)** Chromatogram of affinity coloumn. **(B)** Chromatogram of desalting coloumn. **(C)** Chromatogram of SEC coloumn.

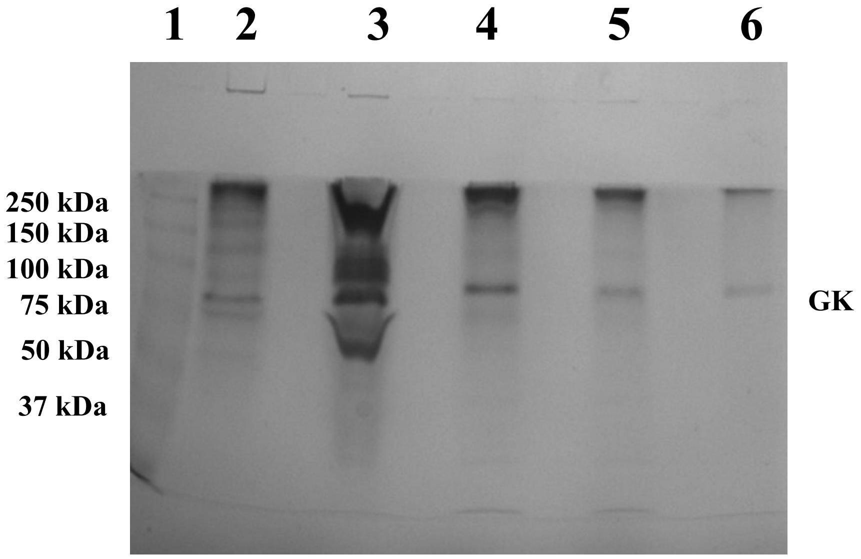

Figure S3. Native-PAGE analysis of recombinant His-GK co-expressed with pKJE7
His-GK co-expressed with pKJE7 was analysed on native-PAGE to examine the dimerization of human GK. Lane 1 - Marker (1.0 µl); Lane 2 - Pellet; Lane 3 - Supernatant; Lane 4 - Affinity column; Lane 5 - Desalting column; Lane 6 - SEC column.
